# Role of penile rehabilitation through daily intake of 5 mg tadalafil on erectile dysfunction after different presentations of penile fracture: a prospective case–control study

**DOI:** 10.1007/s11255-023-03713-y

**Published:** 2023-08-01

**Authors:** Sameh Fayek GamalEl Din, Nashaat Nabil, Mohamed Wael Ragab, Hany Saad, Mariam Labib, Ahmed Abo Sief

**Affiliations:** 1 Andrology and STDs Department, Kasr Alainy Faculty of Medicine, Cairo University, Cairo, Egypt; 2 Andrology and STDs Department, Faculty of Medicine, Beni Suef University, Beni Suef, Egypt; 3 Department of Andrology, Faculty of Medicine, Suez Canal University, Ismailia, Egypt; 4 Egypt Ministry of Health and Population, Cairo, Egypt

**Keywords:** Penile fracture, ArIIEF-5, HADS, 5 mg Tadalafil

## Abstract

**Purpose:**

We aimed in the current study to identify the predictive factors of ED occurrence in healthy individuals following penile fracture surgical repair as well as the effect of penile rehabilitation in the form of daily tadalafil 5 mg intake for 1 month for patients who suffered from ED after penile fracture incident.

**Method:**

The current study was a prospective case–control study. Twenty-five patients were enrolled into the study starting from January (2022) to February (2023). Furthermore, time of presentation was determined, and length of tear intra-operative was measured, and then, a follow-up 1 week postoperatively in the outpatient clinic was conducted. All patients were instructed to start intercourse at least 2 weeks after the first visit provided that the wound epithelialized. Potent patients returned back home. A rehabilitation course of daily tadalafil 5 mg for 1 month was prescribed for patients who started complaining of ED that was confirmed by evaluation with the Arabic validated version of the international index of erectile function (ArIIEF-5). The rehabilitation therapy was terminated by resumption of normal erectile function. Thus, re-evaluation with the ArIIEF-5 was determined according to their response to therapy. Also, the patients were evaluated by hospital anxiety and depression scale (HADS) before and after penile fracture repair.

**Results:**

The current study had demonstrated that a 1% increase in age determines an increase in odds ratio for post-penile fracture ED with 73.6% and 1 cm increase in the length of tear determines an increase in odds ratio for post-penile fracture ED with 20.04 times.

**Conclusion:**

The current study enhances the proper counseling of these patients prior to repairing the defect about the probability of ED occurrence as well as initiating early penile rehabilitation to help these patients resuming their normal sexual activity as soon as possible.

## Introduction

Penile fracture is a rare form of genitourinary trauma, characterized by an acute disruption of the tunica albuginea of the corpus cavernosum. This most commonly occurs in the erect state, when the tunica is both thinner and under increased tension [1]. Delay in presentation is mainly due to fear and embarrassment [2]. At injury, these patients often report a distinct cracking, popping or snapping sound and acute pain followed by detumescence, swelling and discoloration [3]. While penile fracture is an uncommon injury, its incidence is probably underestimated, since patients might not seek medical treatment in emergency rooms due to embarrassment of the clinical situation [4]. Penile fracture may cause erectile dysfunction (ED) that is defined as the inability to achieve or maintain an erection sufficient for satisfactory sexual performance. Other important complications of this injury include penile fibrotic nodules and chordee [5]. It has been reported that the incidence of ED after surgical repair of fracture penis ranges from 0 up to 12% [6]. We aimed in the current study to identify the predictive factors of ED occurrence in healthy individuals following penile fracture surgical repair as well as the effect of penile rehabilitation in the form of daily tadalafil 5 mg intake for patients who suffered from ED after penile fracture incident.

## Patients and methods

The current study was a prospective case–control study. Twenty-five patients were enrolled into the study starting from January (2022) to February (2023). The cases were recruited from andrology outpatient clinic at Beni-Suef University Hospital and Andrology Outpatient Clinic at Kasr Al-Ainy Hospital. The study design, methods and objectives were approved by Beni-Suef Faculty of Medicine Research Ethical Committee (FMBSUREC/09012022) that conforms to Helsinki declaration (2013) [7]. A written informed consent was obtained from each participant prior to sharing in this study. Participants were assured that confidentiality would be maintained during and after the study, and information given would be used only for research purpose.

### Inclusion criteria

Any healthy sexually active male 19–52 years old with penile fracture was enrolled.

### Exclusion criteria

Any male patient with penile fracture on medications affecting sexual function or psychiatric disorder affecting sexual function was excluded. Also, any patient with penile fracture who suffered from chronic medical disease (such as diabetes mellitus and hypertension) or mental illness including depression or personality disorder was excluded.

### All participants were subjected to the following

Personal, sexual and medical histories were obtained from all participants. Furthermore, time of presentation was determined and length of tear intra-operative was measured, and then, a follow-up 1 week postoperatively in the outpatient clinic was conducted to check in any potential postoperative complication. All patients were instructed to start intercourse at least 2 weeks after the first visit provided that the wound epithelialized. Additionally, they are counseled that once any one of them started to complain of ED should contacted us again. Potent patients returned back home. Nine patients out of 25 patients started to complain of ED around 1 month after the first visit that was confirmed by the ArIIEf-5 evaluation. Rehabilitation course of tadalafil 5 mg daily for 1 month was prescribed for all cases who started to complain of ED and would be continued till resumption of normal erectile function. After 1 month of daily 5 mg tadalafil, 5 cases responded and restored their erection as they were re-evaluated by ArIIEF-5, while 4 cases remained suffering from ED. Thus, we resumed the same regimen for the remaining cases till resumption of normal erectile function that was accomplished after another month followed by re-evaluation by the ArIIEF-5 [8]. Also, the patients were evaluated by hospital anxiety and depression scale (HADS) before and after penile fracture repair [9]. At the end of the course, if any patient complained of ED, penile duplex would be performed for him to confirm the diagnosis.

### Statistical analysis

Data were collected, revised, coded and entered to the Statistical Package for Social Science (IBM SPSS) version 20. The qualitative data were presented as number and percentages while quantitative data were presented as mean, standard deviations and ranges when their distribution found parametric.

The comparison between two groups with qualitative data was done using Chi-square test and/or Fisher’s exact test was used instead of Chi-square test when the expected count in any cell was found less than 5. The comparison between two independent groups with quantitative data and parametric distribution was done using independent t test. The confidence interval was set to 95% and the margin of error accepted was set to 5%.

## Results

The current study shows that the average age (years) in patients with penile fracture and ED were 35.89 ± 4.2, while average age (years) in patients with penile fracture and no ED were 25.13 ± 6.24. Furthermore, there were 7 (28.0%) smokers, 2 (8.0%) ex-smokers, and 16 (64.0%) non-smokers (Table 1). Regarding the pattern of tear, 16 (64.0%) cases were corporal, 5 (20.0%) cases were subcoronal, 1 (4.0%) case was corpora and subcoronal, 2 (8.0%) cases were around the neck of the glans penis, and 1 (4.0%) case was bilateral subpubic (Table 1). 56.0% of cases had tunical tear from the right side, while two cases suffered an injury in the urethra and the length of the tear ranged from 1.5 to 5 cm with a mean 2.7 cm (Table 1). There were 16 (64.0%) cases without ED and 9 cases (36.0%) with ED. The ED cases were classified into 3 cases with mild ED, 4 cases with mild-to-moderate ED, and two cases were moderate ED. The mid-shaft was the commonest site of tunical tear as it was reported in 77.8% of cases with ED and 75.0% of cases without ED (Table 1). In the ED group, 22.2% of cases had tunical tear in the right side, while in the no ED group, 75.0% of cases had tunical tear in the right side (Table 1). The average length of tear (cm) in the ED group was 3.64 ± 1.43, while the average length of tear (cm) in the no ED group was 2.17 ± 0.58 (Table 1).

**Table 1:** Comparison between patients with ED and patients without ED regarding special habits, site of tear, site of tunical tear (shaft), side of tunical tear, urethral injury, and length of tear (cm)

	Patients with ED (*N* = 9)	Patients without ED (*N* = 16)	Test value	*P*
Special habits				
Smoker	6 (66.7%)	1 (6.3%)	5.333^b^	0.021*
Ex-smoker	1 (11.1%)	1 (6.3%)		
Non smoker	2 (22.2%)	14 (87.4%)		
Pattern of tear				
Corpora	6 (66.7%)	10 (62.5%)	0.465^a^	0.495
Subcoronal	2 (22.2%)	3 (18.75%)		
Around neck of glans	1(11.1%)	1 (6.25%)		
Bilateral sub pubic	–	1 (6.25%)		
Corpora and subcoronal	–	1 (6.25%)		
Site of tunical tear (shaft)				
Proximal	2 (22.2%)	2 (12.5%)	1.169^a^	0.280
Mid	7 (77.8%)	12 (75.0%)		
Distal	–	2 (12.5%)		
Side of tunical tear				
Right	2 (22.2%)	12 (75%)	9.333^a^	0.002*
Left	3 (33.3%)	4 (25%)		
Bilateral	4 (44.4%)	–		
Urethral injury				
Yes	1 (11.1%)	1 (6.25%)	0.673^a^	1.000
No	8 (88.9%)	15 (93.75%)		
Length of tear (cm)				
Mean ± SD	3.64 ± 1.43	2.17 ± 0.58	− 2.941^b^	0.016*
Range	1.75–5	1.50–3		

^a^Fisher’s exact test

^b^Independent *t* test

In the ED group, all cases suffered from pain, 66.7% of cases presented by the classical eggplant deformity with ecchymosis of the penis, 44.4% of the cases noticed popping sound with sudden detumescence of the erect penis, and 11.1% of the cases presented by localized hematoma (Table 2). While in the no ED group, all cases suffered from pain, 56.3% of the cases presented by the classical eggplant deformity with ecchymosis of penis, 50.0% of the cases noticed popping sound with sudden detumescence of the erect penis, and 12.5% of the cases had Localized hematoma (Table 2). In the no ED group, 16 cases returned back to normal sexual activity immediately after the incident. While the ED Group, 5 cases (55.6%) needed 1 month treatment of daily 5 mg tadalafil to return back to normal sexual activity after the incident and 4 cases (44.4%) needed 2 months treatment of daily 5 mg tadalafil to return back to normal sexual activity after the incident. Moreover, there was statistically significant difference between ED group and No ED group regarding average time to return to sexual activity. Also, the study had shown a statistically significant difference between patients with penile fracture and ED and patients with penile fracture and no ED regarding treatment and follow-up. The current study had demonstrated that the average HADS Score in patients with penile fracture and ED were 10 ± 2.121, while average HADS Score in patients with penile fracture and no ED were 9.00 ± 1.033 (Table 3). In the ED group, 55.6% of them were mild, 33.3% were moderate, and 11.1% were severe, respectively (Table 3). While in the no ED group, 87.5% of them were mild, 12.5% were moderate, and none was severe (Table 3). There were no statistically significant difference between patients with penile fracture and ED and patients with penile fracture and without ED regarding HADS Score (Table 3). Furthermore, our study had shown that 5 cases improved on 5 mg tadalafil intake for 1 month as their mean ArIIEF-5 became (20.22 ± 4.15) (Table 4).

**Table 2:** Comparison between patients with ED and patients without ED regarding clinical presentation

Clinical presentation	Patients with ED		Patients without ED		Test value*	*P*
	No	%	No	%		
Pain	9	100.0	16	100.0	–	–
Classical eggplant deformity with ecchymosis of penis	6	66.7	9	56.3	0.250^a^	0.691
Popping sound with sudden detumescence of erect penis	4	44.4	8	50	0.250^a^	0.691
Localized hematoma	1	11.1	2	12.5	0.010^a^	1.000

^a^Fisher’s exact test

**Table 3:** Comparison between patients with ED and patients without ED regarding HADS Score

	Patients with ED (*N* = 9)	Patients without ED (*N* = 16)	Test value	*P*
HADS score				
Mild	5 (55.6%)	14 (87.5%)	− 3.640^a^	0.056
Moderate	3 (33.3%)	2 (12.5%)		
Severe	1 (11.1%)	–		
Mean ± SD	10 ± 2.121	9.00 ± 1.033	1.328^b^	0.213
Range	8–13	8–11		

^a^Fisher’s exact test

^b^Independent *t* test

*HADS* hospital anxiety and depression scale

**Table 4:** ArIIEF-5 score after 1 month of treatment and ArIIEF-5 score after 2 months of treatment

	IIEF-5 after 1 month treatment (*N* = 5 patients responded)	IIEF-5 after 2 month treatment (*N* = 4 patients responded)
Mean	20.22	20.25
Median	22.00	21.50
Std. Deviation	± 4.15	± 2.87
Minimum	13.00	16.00
Maximum	25.00	22.00

N.B. Arabic validated version of the international index of erectile function (ArIIEF-5)

While, 4 cases needed another month of 5 mg tadalafil to improve as their mean ArIIEF-5 became (20.25 ± 2.87) (Table 4). In a multiple logistic regression model for post-penile fracture ED, age and length of tear (cm) were the only significant independent variables (*p = *0.046, *p = *0.04, *p < *0.05, respectively) (Table 5). As shown in Table 5, considering EXP(β) for the odds ratio evaluation, with a 95% degree of confidence, a 1% increase in age determines an increase in odds ratio for post-penile fracture ED with 73.6% and 1 cm increase in the length of tear determines an increase in odds ratio for post-penile fracture ED with 20.04 times. Meanwhile, there was non-significant association between post-penile fracture ED and special habits and pattern of tear (*P > *0.05).

**Table 5:** Logistic regression analysis of the relationship between post-penile fracture ED& study variables

	*B*	S.E.	Wald		df	Sig.	Exp(B)	95% C.I for EXP (B)	
								Lower	Upper
Age years	0.551	0.276		3.989	1	0.046*	**1.736**	1.010	2.981
Length of tear (cm)	2.998	1.463		4.198	1	0.040*	**20.041**	1.139	352.629
Special habits				1.172	2	0.557			
Special habits (1)	− 16.325	40,192.975		0.000	1	1.000	0.000	0.000	
Special habits (2)	− 11.709	40,192.975		0.000	1	1.000	0.000	0.000	
Pattern of tear				0.694	3	0.875			
Pattern of tear (1)	11.698	40,192.975		0.000	1	1.000	120,386.804	0.000	
Pattern of tear (2)	14.600	40,192.975		0.000	1	1.000	2,190,486.529	0.000	
Pattern of tear (4)	− 1.103	56,841.447		0.000	1	1.000	0.332	0.000	

## Discussion

The current study demonstrated that there were 7 (28.0%) smokers, 2 (8.0%) ex-smokers, and 16 (64.0%) non-smokers, and the mean age in the ED group was 35.89 ± 4.2 years. Similarly, Sharma et al. (2021) reported that the mean age at presentation was 33.64 ± 9.46 years [10]. In contrast, Altan et al. (2022) found that the median age of patients was 46 years [11]. Regarding the side of the tear, 56.0% of cases had tunical tear on the right side and the length of the tear ranged from 1.5 to 5 cm with mean 2.7 cm. The average defect size detected on ultrasound was 7 mm, which was much smaller than the actual size of the defect on surgical exploration (median 1.5 cm). Also, all cases in the current study demonstrated unilateral tears, mostly transverse with a length ranged 0.5–4 cm going with findings reported by De Rose et al. (2001) [12]. The right corpora cavernosa was the commonest side affected in the current study (57.3%) which is in line with that reported in the previous studies [13–15].

Ateyah et al. (2008) explained the fact that most patients are right-handed, and during manipulation of penis, it usually bends toward left, resulting in tear on the right side [14]. While some authors reported that the distal third of the penile shaft was the most often involved site [16]. Only two cases suffered an injury in the urethra in the current study. Similarly, Altan et al. (2022) reported that two patients had concomitant urethral injury [11]. While Sharma et al. (2021) reported urethral injury 3 (4.4%) patients of their series [10]. Amer et al. (2016) suggested that a urethral injury should be suspected in patients with gross hematuria, microscopic hematuria, or urinary retention, and they reported the incidence of urethral injury with penile fracture 6.1% [17]. Sharma et al. (2021) reported that urethral injury was suspected in six patients who presented with blood at the meatus and all of them underwent retrograde urethrogram [10]. Furthermore, the incidence of urethral injury ranged from 0 to 3% in Iran, the Arabic Gulf countries and Japan, while in the European countries, it ranged from 20 to 38% [18, 19]. Trauma during sexual intercourse was reported as the main cause of penile injury in America, taghaandan a practice that meant manipulating the erect penis in an attempt to achieve detumescence was reported as the major cause in the Middle East [20]. Rolling over an erect penis in bed and masturbation were the most common causes in Japan [21]. Regarding the causes of penile fracture in the current study, there were 16 (64.0%) cases due to vigorous sexual intercourse, 4 (16.0%) cases due to masturbation, 3 (12.0%) cases due to direct trauma and 2 (8.0%) due to rolling over the erect penis. Consistently, Amer et al. (2016) found sexual intercourse as the main cause of penile fracture in 46% of the patients, followed by forced flexion (21%) and masturbation (18%) in a pooled data of over 3,000 patients [17]. Also, Sharma et al. (2021) reported that the most common cause was sexual intercourse (78%) [10].

The current study showed that the clinical presentation was mainly pain as reported in all cases followed by the classical eggplant deformity with ecchymosis of penis as reported in 15 (60.0%) cases then popping sound with sudden detumescence of erect penis as reported in 12 (48.0%) cases. In contrast, Sharma et al. (2021) reported that 53% of the patients reported a popping sound and a sudden detumescence, 80.8% of the patients presented with typical eggplant deformity with diffuse ecchymosis, and 19.1% of the patients presented with a localized hematoma [10]. Moreover, Altan et al. (2022) reported that the most common findings are penile hematoma, swelling and penile deviation [11]. The present study showed that the time of presentation and surgical repair were within 24 h for all patients. Similarly, De Luca et al. (2017) stated that penile fracture required early diagnosis and repair [22]. In contrast, Sharma et al. (2021) reported that erectile function of patients with delayed presentation compared favorably with those who presented immediately after trauma with no statistically significant difference [10]. In the current study, the majority of the patients returned to normal sexual activity after 1–2 weeks, while 5 cases do so after 1 month and 4 cases do so after 2 months, respectively. It has been shown that spontaneous recovery of sexual function could occur up to 2 years after injury [23]. Although 64% of those men who recover from impotence do so within 12 months [24], yet the preferable timing of assessing sexual function is 24 months post-injury [25]. The current study revealed that there were 16 (64.0%) cases without ED, while 9 cases (36.0%) suffered from ED. They were 3 cases with mild ED, 4 cases with mild-to-moderate ED, and two cases were moderate ED, respectively. Additionally, the present study showed that there were 16 (64%) cases who did not receive any treatment, while 9 (36%) received treatment tadalafil (5 mg). Five of them (20%) required treatment and follow-up for 1 month, while 4 (16%) cases required treatment and follow-up for two months.

Similarly, Altan et al. (2022) reported that ED was present in 13 patients (52%) [11]. In contrast, Sharma et al. (2021) reported good functional outcome in 75.8% of the cases with no complications [10]. Our study revealed that there was statistically significant increasing age among patients with penile fracture and ED than patients with penile fracture and without ED. It has been repeatedly reported that older patients are at greater risk of developing ED after penile fracture with urethral injury, albeit without giving an explanation [26]. Our study showed that there were highly statistically significant difference between patients with penile fracture and ED than patients with penile fracture and without ED regarding side of tunical tear and length of tear. Sharma et al. (2021) reported that patients with unilateral right or left corporal involvement had 8.5% and 4.2% incidence of ED, respectively; whereas the risk of developing ED was found to be statistically significant in bilateral corporal involvement [10]. The reason behind it may be explained by the fact that veno-occlusive dysfunction might be a coexistent pathology at the fracture site which increased by twofold in case of bilateral tears. In the aforementioned study, the mid-shaft was the most common location, because this is the weakest part of the tunica during erection. Urethral injury was found in 3 (4.4%) patients, but erectile function was preserved on long-term follow-up. This could be seen in line with our findings as ED was not reported in cases associated with urethral injury. All the tunical tears in the aforementioned study were on the ventral aspect of the penis, because the dorsal aspect is supported by neurovascular bundles with thick tunica albuginea, which is less liable to stretch beyond its limit during erection [27]. In contrast, Ateyah et al. (2008) demonstrated that the proximal third of the penile shaft is the most common site of injury [14].

Remarkably, the current study had shown that aging and length of the tear are the two most important determinant factors for occurrence of ED following penile fracture that necessitates early penile rehabilitation in the form of daily tadalafil 5 mg that should be supplemented till restoration of normal sexual activity. In the same context, Ortac et al. (2020) delivered out similar results [28]. In contrast, Sharma demonstrated that age and bilateral corporal tear were the most determinant factors for ED development [10]. To wrap up, the current study is one of the first to demonstrate the beneficial effect of daily administration of 5 mg tadalafil to patients with penile fracture especially older age and patients with large-sized penile tear as they were at higher risk of developing ED. Admittedly, the small sample size is the major limitation of the current study. However, collecting penile fracture cases is a difficult task and penile fracture is a rare emergency condition [22]. Furthermore, in ability to follow the patients up for longer duration is another limitation. However, we tied the follow-up period with the response to tadalafil 5 mg daily which made it as long as it took for the patients to respond to our regimen and restored their normal sexual activity. Finally, we evaluated the anxiety and depression statuses of the patients using non-Arabic validated version can be added to the limitations of the study.

## Conclusion

The current study affirms the negative impact of age and the size of the penile tear on the erectile function for patients with penile fracture. Thus, it enhances the proper counseling of these patients prior to repairing the defect about the probability of ED occurrence as well as initiating early penile rehabilitation to help these patients resuming their normal sexual activity as soon as possible.

## Data Availability

The data that support the findings of this study are available from the corresponding author upon reasonable request.
